# New Horizons in Diabetology

**DOI:** 10.1155/2016/9075924

**Published:** 2016-01-18

**Authors:** Ali Tootee, Garth Warnock, Aziz Ghahari, Bagher Larijani

**Affiliations:** ^1^Diabetes Research Center, Endocrinology and Metabolism Clinical Sciences Institute, Tehran University of Medical Sciences, Tehran, Iran; ^2^University of British Columbia, Vancouver, Canada; ^3^Vancouver General Hospital, Vancouver, Canada; ^4^Endocrinology and Metabolism Research Center (EMRC), Tehran University Medical Sciences, Tehran, Iran

The prevalence of diabetes has alarmingly increased in both developed and developing countries all across the world in the recent years. The prevalence of different complications and comorbid conditions associated with diabetes is also rampantly increasing, thereby negatively affecting lives of many people. In fact, obesity which is associated with diabetes is considered as a major public health concern in many countries. Nevertheless, it can be argued that still more progress is to be made in different fields of prevention, diagnosis, and treatment of diabetes. This gap in the science of Diabetology, arguably, stems from insufficient synchronized interdisciplinary basic and clinical research in the field. This deficiency may be addressed through finding the gaps in the published literature by means of plotting different diabetes maps and focusing research on them to promote the concept of translational medicine.

Lack of comprehensive diabetes research maps and the scarcity of the literature in the related interdisciplinary fields can be considered as major hindrance to the future advancement of the science of Diabetology. With this view, we conceived the concept of publication of a special issue on the subject and invited authors to submit any original research or review paper with the potential of opening new horizons to prevention, screening, and treatment of diabetes. Out of approximately 50 manuscripts submitted, in this special issue, 15 articles have been approved by reviewers to be published on a wide range of relevant basic and clinical subjects.

In the field of screening for diabetes, findings of a study by S. M. Joyce-Tan et al. demonstrated that genetic variants of the RAS can modestly influence the risk of type 2 diabetes. P. Haghvirdizadeh et al., in a different study, reported that R219K polymorphism of ABCA1 gene could be considered as a genetic risk factor for type 2 diabetes among certain populations. In another article, M. Khodaeian et al. assert that genome wide association studies on large number of samples can be helpful in the identification of diabetes susceptible genes and may be used as an alternative to studying individual candidate genes which is a time-consuming and expensive method. In a different article, P. Haghvirdizadeh et al. findings provide evidence for the contribution of common KCNJ11 genetic variants to the development of DM.

In regard to the underlying mechanisms of diabetes and its complications, M. Káplár et al., in an original research article, demonstrated a dual role for mannose binding lecithin as a risk factor for carotid intima media thickness in patients with type 2 diabetes mellitus. N. Bertoncello et al., in another article, shed some light on the complex underlying mechanisms involved in the development of diabetic nephropathy.

As for novel diagnostic, preventive, and therapeutic options for diabetes, T. Mizushige showed that urinary angiotensinogen could be a prognostic marker of renoprotective effects of alogliptin in patients with type 2 diabetes. Findings of a study by R. Díez-Láiz demonstrate significant therapeutic potentials for* Plantago ovata* husk as an oral antihyperglycemic agent for treatment of type 2 diabetes. An article by M. S. Klein and J. Shearer argues that metabolomics has the potential to enable informed decision-making in the realm of personalized medicine. S. J. Hashemian et al., in a comprehensive review article, claim that despite all unresolved concerns about clinical applications of mesenchymal stem cells, this group of stem cells still remains a promising therapeutic modality for treatment of diabetes. A study by K. Kurek et al. demonstrates that myriocin can find potential future application as a therapeutic agent for the reduction of insulin resistance and its serious consequences in obese patients. Findings of S. Wang et al. study show that certain renoprotective therapeutic agents which can specifically abolish CTGF CCN2 expression, or nonspecifically inhibit CTGF CCN2 expression, may be protective against the development and progression of diabetes nephropathy. P. Senesi et al., in their study, demonstrate that metformin treatment can prevent sedentariness related damage in mice. In this regard, in another study by M. Bo et al., it is asserted that an inappropriate and aggressive glucose lowering therapeutic approach in frail and vulnerable elderly residents of long-term care facilities can adversely affect their health.

It can be concluded that this special issue provides a series of original and review articles on a wide range of diabetes-related subjects which can assist researchers to design and carry out research projects in similar directions in the future. Moreover, this issue may be helpful in spotting current gaps in the science of Diabetology and facilitate focusing more research on them. We hope that the present special issue provides useful information for a superior understanding of the underlying mechanisms involved in the pathogenesis of diabetes as well as novel diagnostic and therapeutic techniques for the diseases.

